# Meta-analysis based gene expression profiling reveals functional genes in ovarian cancer

**DOI:** 10.1042/BSR20202911

**Published:** 2020-11-19

**Authors:** Lin Zhao, Yuhui Li, Zhen Zhang, Jing Zou, Jianfu Li, Ran Wei, Qiang Guo, Xiaoxiao Zhu, Chu Chu, Xiaoxiao Fu, Jinbo Yue, Xia Li

**Affiliations:** 1 Department of Oncology, Institute of Basic Medicine, The First Affiliated Hospital of Shandong First Medical University, No. 18877 Jingshi Road, Jinan 250062, China; 2 Department of Outpatient, Shandong Cancer Hospital and Institute, Shandong First Medical University and Shandong Academy of Medical Sciences, No. 440, Ji Yan Road, Jinan 250117, China; 3 Affiliated Hospital of Shandong University of Traditional Chinese Medicine, No. 42 Wenhua Xi Road, Jinan 250011, China; 4 Surgical Planning Laboratory, Department of Oral and Maxillofacial Surgery, Houston Methodist Research Institute, Houston, TX, U.S.A.; 5 Department of Radiation Oncology, Shandong Cancer Hospital and Institute, Shandong First Medical University and Shandong Academy of Medical Sciences, No. 440, Ji Yan Road, Jinan 250117, China

**Keywords:** Differentially expressed genes, Gene expression omnibus, Microarray analysis, Ovarian cancer

## Abstract

**Background:** Ovarian cancer causes high mortality rate worldwide, and despite numerous attempts, the outcome for patients with ovarian cancer are still not well improved. Microarray-based gene expressional analysis provides with valuable information for discriminating functional genes in ovarian cancer development and progression. However, due to the differences in experimental design, the results varied significantly across individual datasets.

**Methods:** In the present study, the data of gene expression in ovarian cancer were downloaded from Gene Expression Omnibus (GEO) and 16 studies were included. A meta-analysis based gene expression analysis was performed to identify differentially expressed genes (DEGs). The most differentially expressed genes in our meta-analysis were selected for gene expression and gene function validation.

**Results:** A total of 972 DEGs with *P*-value < 0.001 were identified in ovarian cancer, including 541 up-regulated genes and 431 down-regulated genes, among which 92 additional DEGs were found as gained DEGs. Top five up- and down-regulated genes were selected for the validation of gene expression profiling. Among these genes, up-regulated CD24 molecule (*CD24*), SRY (sex determining region Y)-box transcription factor 17 (*SOX17*), *WFDC2*, epithelial cell adhesion molecule (*EPCAM*), innate immunity activator (*INAVA*), and down-regulated aldehyde oxidase 1 (*AOX1*) were revealed to be with consistent expressional patterns in clinical patient samples of ovarian cancer. Gene functional analysis demonstrated that up-regulated *WFDC2* and *INAVA* promoted ovarian cancer cell migration, *WFDC2* enhanced cell proliferation, while down-regulated *AOX1* was functional in inducing cell apoptosis of ovarian cancer.

**Conclusion:** Our study shed light on the molecular mechanisms underlying the development of ovarian cancer, and facilitated the understanding of novel diagnostic and therapeutic targets in ovarian cancer.

## Background

Ovarian cancer is the second most encountered gynecologic cancer worldwide with high mortality and low rate of 5-year survival due to the difficulty in early diagnosis and high rates of recurrence and metastasis [1]. If ovarian cancer could be diagnosed at an early stage, 90% patients could survive for more than 5 years with appropriate treatment [2]. Despite numerous attempts, the common molecular events for early diagnosis of ovarian cancer remain to be established. Gene expression profile analysis is a powerful research strategy, which integrates data in genetics, molecular transcription, and functional genomics to reveal dysregulated genes between patients and healthy donors. Microarray provides increasing body of gene-wide transcriptional data regarding ovarian cancer. For example, Moreno et al*.* proposed that cellular proliferation, cell cycle, DNA damage, and apoptosis were up-regulated in ovarian cancer by using microarrays [3]. Bowen et al. identified alterations in expression levels of gene products functioning in several signaling pathways including Wnt, Notch, TGFβ/BMP, and canonical cell cycle in ovarian cancer by microarray analysis [4]. However, results vary between studies due to the diversity in cohort selection, specimen source, and experimental designs. Combining different microarray datasets is advantageous to enhance statistical power to detect the dysregulated genes that might be functional in ovarian cancer development and progression.

Microarray data integration-based meta-analyses depend on efficient *in silico* tools. With the advances of ever-growing theories and bioinformatics tools, we can now employ *in silico* tools to efficiently combine multiple microarray datasets ignoring different populations, experimental designs, and different specimen sources [5–7]. NetworkAnalyst is a useful web-based tool, which functions in many aspects such as preliminary data processing, sample annotation, batch effect adjustment, dataset integration, and results visualization [8–12]. To maximally overcome the impact caused by the differences in study design and platform usage among different datasets, ‘Combining Effect Size (ES)’ analysis and Random Effect Modeling (REM) were applied to achieve more consistent and accurate results by taking into consideration both direction and magnitude of gene expression changes. In the present study, 16 eligible microarray datasets for ovarian cancer were selected from publicly available dataset repositories. Totally, 972 differentially expressed genes (DEGs) with *P*-value < 0.001 were identified, including 541 up- and 431 down-regulated genes, respectively. Interestingly, by comparing the list of meta-based DEGs to that of individual microarray dataset, 92 additional DEGs were demonstrated as gained DEGs. Expression profiles for the CD24 molecule (*CD24*), SRY (sex determining region Y)-box transcription factor 17 (*SOX17*), WAP four-disulfide core domain 2 (*WFDC2*), the epithelial cell adhesion molecule (*EPCAM*), innate immunity activator (*INAVA*), and aldehyde oxidase 1 (*AOX1*) were validated in clinical cancer samples of ovarian cancer, which were among top five dysregulated DEGs revealed by our meta-analysis. Functional analysis uncovered that *WFDC2, INAVA*, and *AOX1* affect cell proliferation, migration, and apoptosis, respectively. Our results provided with novel molecular mechanisms for the development and progression of ovarian cancer and propose potential targets for the prevention and treatment for clinics [13].

## Methods

### Materials identification and selection of eligible gene expression datasets for meta-analysis

**Figure 1:**
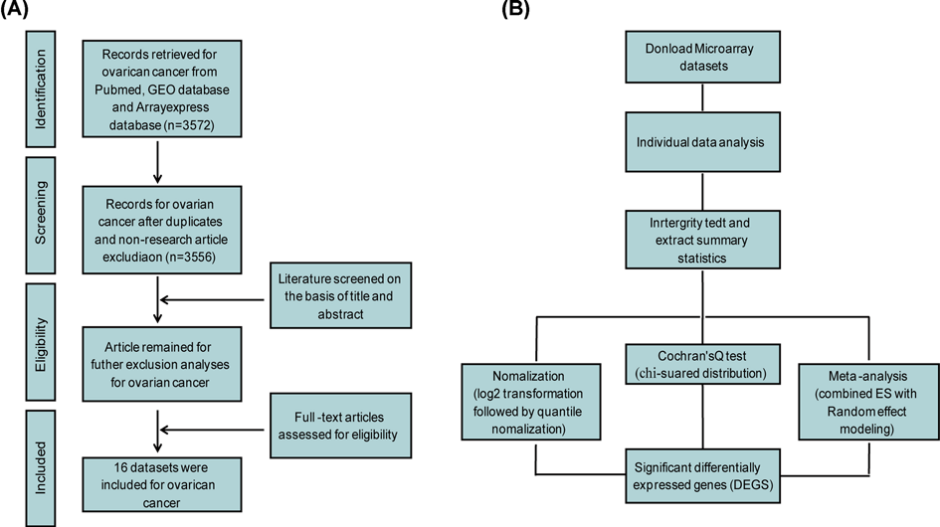
Flowcharts for microarray datasets selection and meta-analysis (**A**) Selection process of microarray datasets for meta-analysis of gene expressional signature in ovarian cancer. (**B**) Process of meta-analysis based data exploration.

Microarray-based gene expression profiling studies for ovarian cancer were identified in the PubMed database (http://www.pubmed.gov), Gene Expression Omnibus (GEO, https://www.ncbi.nlm.nih.gov/gds/) and ArrayExpress dataset of the European Molecular Biology Laboratory-European Bioinformatics Institute (http://www.ebi.ac.uk/arrayexpress/). The following keywords were used: (‘Ovarian neoplasms’ [Mesh]) and (microarray or gene profile or gene profiling). Eligible studies and datasets should follow these inclusive criteria: (1) case and control studies of human; (2) analysis of gene expression profiling; (3) comparable experimental conditions and untreated; (4) available complete raw and processed microarray data. Studies were excluded if they were: (1) letters, abstracts, meta-analysis, review articles, and human case reports; (2) cell lines used in experimental design; (3) RT-PCR only for profiling studies; (4) studies without healthy controls. All the datasets and references, which conformed to the above-mentioned criteria, were manually screened. The complete workflow is shown in Figure 1 for eligible dataset selection. The latest search was performed on 15 March 2019.

### Data extraction and processing

Full text and supplementary materials of selected articles were extracted from each identified study: GEO series accession number, number of patients and healthy donors, specimen sources, platform of microarray and references. The series matrix files were downloaded from GEO datasets for all studies, and common Entrez IDs were used to substitute all the gene probes in accordance with the corresponding microarray platforms, with the exception of GSE17308, in which the gene probe was substituted by Genbank ID. Before integrative meta-analysis, individual dataset was normalized by R-mediated mean, log2 transformation and quantile normalization. Expression data of patients with ovarian cancer and healthy donors were defined as class 2 and class 1, respectively, according to the guidelines of NetworkAnalyst [6].

### Batch effect adjustment and individual data analysis

Batch effect correction option in NetworkAnalyst was used to reduce potential study-specific batch effects, by which the normalized individual datasets were subjected to the well-established ComBat procedures [11]. Emperical Bayes methods were used to stabilize the expression ratios for genes with very high or very low ratios, stabilize gene variances by shrinking variances across all other genes, and protect their inference from artifacts in the data. To compare the sample clustering patterns with and without applying the ComBat procedures, the results were visually examined using the principal component analysis (PCA).

### Meta-analysis

We conducted the meta-analysis using NetworkAnalyst, a web interface for integrative statistical analysis and visualization. In the option of ‘multiple gene expression data’, all the datasets were uploaded to the ‘multiple gene expression data’ input area and analyzed in a streamlined manner, including data processing for Entrez ID/Genbank ID, annotation check, and PCA plot examining, confirmation of data normalization, individual DEG analysis and data summary. For NetworkAnalyst-based DEGs discrimination, the cutoff of *P*-value was adjusted to 0.001. Furthermore, all datasets were subjected to integrity check to ensure that the merged data could be carried out by ES combination, which would generate more biologically consistent meta-based DEGs by incorporation of both the magnitude and direction of gene fold change. While performing Cochran’s Q test on the integration data, the random-effects model was suggested to be used when Q values deviate significantly from a chi-squared distribution; otherwise, the fixed-effects model was selected. The Q values deviate significantly from a chi-squared distribution of the current study, so the present study used REM which assumes that each study contains a random effect size that could incorporate unknown cross-study heterogeneities, as demonstrated by Cochran’s Q tests [10,12]. ‘Define custom signature’ tool from NetworkAnalyst was used to produce the heatmap visualization for both top 25 up- and down-regulated genes.

### Patients and specimens

Thirty epithelial ovarian carcinoma samples (cancer tissues) (Supplementary Table S1) and thirty unpaired normal ovary samples (paracancer tissues) were collected at the Shandong Cancer Hospital and Institute, Shandong First Medical University and Shandong Academy of Medical Sciences. No local or systemic neo-adjuvant radiotherapy and/or chemotherapy and targeted therapy had been received. Written informed consent was obtained from each patient. Our study was approved by the Ethics Committee of Shandong First Medical University and Shandong Academy of Medical Sciences.

### Cell culture and siRNA transfection

OVCAR-3 cell line was obtained from Procell Life Science & Technology Co., Ltd (Wuhan, China). HOSEpiC, and CAOV-3 cell was purchased from BeNa culture collection (Beijing, China). CAOV-3 cells were maintained in Dulbecco’s Modified Eagle’s Medium (DMEM) (Bioind, Kibbuiz, Israel). OVCAR-3 cells were cultured in Roswell Park Memorial Institute (RPMI) 1640 Medium (Bioind, Kibbuiz, Israel). All culture media were supplemented with 10% Fetal Bovine Serum (FBS) (Bioind, Kibbuiz, Israel) and 1% penicillin/streptomycin. Cells were cultured in a humidified incubator at 37°C and 5% CO_2_. siRNAs for *CD24, SOX17, WFDC2, EPCAM, INAVA*, basonuclin 1 (*BNC1*), *AOX1*, gasdermin E (*GSDME*), receptor accessory protein 1 (*REEP1*), and heart and neural crest derivatives expressed 2 (*HAND2*) were transfected into OVCAR-3 and CAOV-3 cells by HiperFect at the concentration of 50 nM according to the manufacturer’s instructions. The sequences of siRNAs used for *CD24, SOX17, WFDC2, EPCAM, INAVA*, and *AOX1* knockdown are listed in Supplementary Table S2. The sequences of primers used for *CD24, SOX17, WFDC2, EPCAM, INAVA, BNC1, AOX1, GSDME, REEP1*, and *HAND2* are listed in Supplementary Table S3.

### Real-time quantitative RT-PCR

Total RNA was extracted by using TRIzol reagent (Invitrogen, Carlsbad, U.S.A.) according to the manufacturer’s instructions. RNA was reverse transcribed with the mRNA First-Strand cDNA Synthesis Kit (Vazyme, Nanjing, China) for mRNA. Quantitative RT-PCR (qRT-PCR) assay was performed on an Applied Biosystems 7500 instrument (Applied Biosystems, Foster, U.S.A.) by using SYBR Green (Invitrogen, Carlsbad, U.S.A.). Relative RNA quantification was calculated via the comparative 2^−ΔΔ*C*_t_^ method. The relative expression of indicated genes was normalized to that of GAPDH in each sample.

### Cell apoptosis assay

For apoptosis assay, cells were seeded into 12-well plates and allowed to adhere overnight. Then, the cells were digested with EDTA-free trypsin and left to dissociate into single cells. After being washed with PBS, the harvested cells were resuspended at a density of 1 × 10^6^ cells/ml in 1× binding buffer and double stained with FITC Annexin V and Propidium Iodide using FITC Annexin V Apoptosis Detection Kit I (BD Biosciences, U.S.A.). A minimum of 1 × 10^4^ single-cell events were acquired by flow cytometry (BD FACSVerse, U.S.A.), and cell apoptosis was analyzed by using FlowJo software.

### Cell proliferation assay

OVCAR-3 and CAOV-3 cells were seeded at 2000 cells per well in 96-well plates after transfection. Cell Counting Kit-8 (CCK8) assay was used to measure cell number and viability, and the absorbance was determined using a spectrophotometric plate reader at 450 nm (SPECTRAMAX 190, U.S.A.). The data were represented as mean ± SD. Each experiment was repeated three times.

### Cell migration assays

Cell migration was assessed by transwell experiments. For cell migration assay, indicated cells were trypsinized, re-suspended in serum-free DMEM, and placed in the upper chamber (4 × 10^4^ cells/well). Then, DMEM supplemented with 10% FBS was added to the lower chamber. The plates were incubated for 48 h. Cells in the upper chamber were completely removed with a cotton swab. Cells migrating into the lower chamber were washed with PBS, fixed in 4% paraformaldehyde, and stained with 0.5% Crystal Violet. Finally, the cells were counted under a microscope in five random fields.

### Statistical analysis

The web-server NetworkAnalyst analyzed the data and performed meta-analysis by REM-based combined ES statistics. The present study adjusted *P*-value < 0.001 to discriminate the DEGs. All experiments were performed at least three independent times, unless otherwise stated. Values are presented as the mean ± SD. The two-tailed Student’s *t* test was used for comparisons between two groups. GraphPad 8.0 was used for statistical analysis of data and data plotting. *P*-value ≤0.05 was considered significant.

## Results

### Selection of eligible microarray datasets

A total of eligible 16 microarray datasets were selected for ovarian cancer (GSE26712, GSE40595, GSE66957, GSE18520, GSE27651, GSE6008, GSE38666, GSE17308, GSE14407, GSE14001, GSE29220, GSE29450, GSE52037, GSR15578, GSE36668 and GSE23391) [1,4,14–25], respectively, as described in ‘Methods’ section (Figure 1A). All the 16 datasets were screened according to the inclusion and exclusion criteria. A total of 135/505 (healthy controls/patients) samples were included in the present study. Four microarray platforms were applied in the selected datasets, including Affymetrix Human Genome U133 Plus 2.0 Array, Affymetrix Human Genome U133A Array, Rosetta/Merck Human RSTA Custom Affymetrix 2.0 microarray, and PC Human Operon 21k v2. In addition, various tissues, such as stroma, epithelium, mucinous, clear cell, and cell-free saliva, were used for the microarray analysis. In the process of data integration, patient samples from different sources were not distinguished, for the purpose to reveal the common gene signature in ovarian cancer. Table 1 presents the detailed information for each dataset, including GEO accession number, sample composition, sample source, and the corresponding references.

**Table 1:** Characteristics of each selected microarray dataset for the meta- analysis

GEO accession number	Sample (Ctl/Pt)	Sample source	Platform	Reference
GSE26712	*n*=195 (10/185)	Ovarian tissue	Affymetrix Human Genome U133A Array	[19]
GSE40595	*n*=77 (14/63	Ovarian tissue	Affymetrix Human Genome U133 Plus 2.0 Array	[14]
GSE66957	*n*=69 (12/57	Ovarian tissue	Rosetta/Merck Human RSTA Custom Affymetrix 2.0 microarray	Unavailable
GSE18520	*n*=63 (10/53)	Ovarian tissue	Affymetrix Human Genome U133 Plus 2.0 Array	[15]
GSE27651	*n*=49 (6/43)	Ovarian tissue	Affymetrix Human Genome U133 Plus 2.0 Array	[16]
GSE6008	*n*=103 (4/99)	Ovarian tissue	Affymetrix Human Genome U133A Array	[20]
GSE38666	*n*=45 (20/25)	Ovarian tissue	Affymetrix Human Genome U133 Plus 2.0 Array	[1]
GSE17308	*n*=32 (4/28)	Ovarian tissue	PC Human Operon 21k v2	[17]
GSE14407	*n*=24 (12/12)	Ovarian tissue	Affymetrix Human Genome U133 Plus 2.0 Array	[4]
GSE14001	*n*=23 (3/20)	Ovarian tissue	Affymetrix Human Genome U133 Plus 2.0 Array	[21]
GSE29220	*n*=22 (11/11)	Cell-free saliva	Affymetrix Human Genome U133 Plus 2.0 Array	[18]
GSE29450	*n*=20 (10/10)	Ovarian tissue	Affymetrix Human Genome U133 Plus 2.0 Array	[22]
GSE52037	*n*=20 (10/10)	Ovarian tissue	Affymetrix Human Genome U133 Plus 2.0 Array	[23]
GSE15578	*n*=17 (6/11)	Ovarian tissue	Affymetrix Human Genome U133 Plus 2.0 Array	Unavailable
GSE36668	*n*=12 (4/8)	Ovarian tissue	Affymetrix Human Genome U133 Plus 2.0 Array	[24]
GSE23391	*n*=4 (1/3)	Ovarian tissue	Affymetrix Human Genome U133 Plus 2.0 Array	[25]

The table lists the datasets of 16 GEO samples used in the present study, the samples are mainly from ovarian tissue and cell-free saliva. A total of 505 patient samples and 135 controls are obtained. The platforms used mainly include Affymetrix Human Genome U133A Array, Affymetrix Human Genome U133 Plus 2.0 Array, Rosetta/Merck Human RSTA Custom Affymetrix 2.0 Microarray, and PC Human Operon 21K V2, and the corresponding references are also listed in the table.

### Identification of common DEGs in ovarian cancer

**Figure 2:**
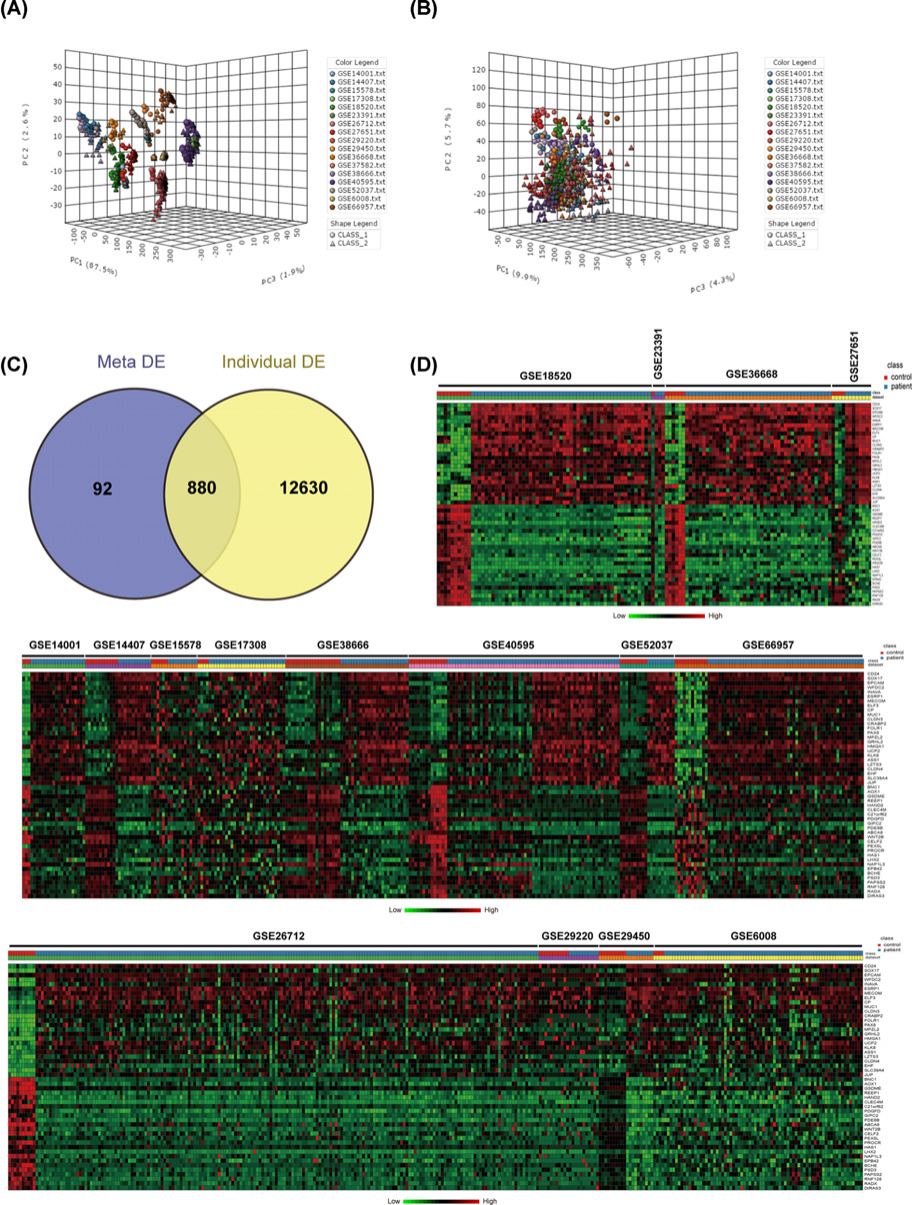
DEGs identified by meta-analysis (**A**) PCA-3D plot for sample clustering of microarray datasets without batch effect adjustment. (**B**) PCA-3D plot for sample clustering of microarray datasets after batch effect adjustment. (**C**) Venn diagram of DEGs by meta-analysis (Meta-DE) and individual microarray dataset analysis (Individual-DE). (**D**) Heat-map visualization of expressional profiles for top 25 up- and down-regulated DEGs identified by meta-analysis. Genes were ranked by combined ES value, and the representative heat-map was generated by ‘Define Custom Signatures’ tool of NetworkAnalyst. Class of red: healthy donor; class of green: patient.

The workflow for meta-analysis in the present study was illustrated in Figure 1B. To identify the transcriptional signature between healthy donors and patients, a total of 16 datasets were simultaneously analyzed by NetworkAnalyst. The 16 datasets were uploaded in succession, and each dataset was processed by gene ID matching, sample (control/patient) annotation, and individual DEGs identification. To remove batch effects among different datasets, ‘ComBat’-based batch effects adjustment were performed [26], and the results with and without the adjustment were visualized by PCA plots, respectively (Figure 2A,B). All the 16 gene expressional microarray data were then integrated and merged. Meta-analysis was conducted following the Cochran’s Q test REM and ES statistical methods, which facilitated to reveal the DEGs between healthy donors and patients across different microarray datasets by permitting variable true effect size and integrating unknown cross-study heterogeneities. We found a total of 972 DEGs, including 541 up- and 431 down-regulated genes across the 16 datasets with significance threshold of adjusted *P*-value < 0.001. Besides, by taking the advantage of meta-analysis, there were 92 gained genes with consistent expressional profiles and 12638 lost genes with inconsistent expressional changes across datasets acquired by comparing meta-based DEGs with that of individual gene expression analysis (Figure 2C). Expression profile of the top 25 up- and down-regulated genes among the 972 identified DEGs was visualized by heatmap (Figure 2D). *CD24, SOX17*, and *EPCAM* were among the most significantly up-regulated genes, while *BNC1, AOX1*, and *GSDME* were among the most significantly down-regulated genes (Table 2).

**Table 2:** Top 20 DEGs identified by meta-analysis across different datasets

EntrezID	Gene symbol	Gene name	Combined ES	Adjusted *P*-value
Top 10 overexpressed genes				
100133941	*CD24*	CD24 molecule	2.8049	7.02E-08
64321	*SOX17*	SRY (sex determining region Y)-box transcription factor 17	2.6537	5.12E-06
4072	*EPCAM*	epithelial cell adhesion molecule	2.5631	4.70E-07
10406	*WFDC2*	WAP four-disulfide core domain 2	2.4890	1.08E-06
55765	*INAVA*	innate immunity activator	2.3726	9.61E-07
54845	*ESRP1*	epithelial splicing regulatory protein 1	2.2399	6.31E-06
2122	*MECOM*	MDS1 and EVI1 complex locus	2.2230	7.31E-07
1999	*ELF3*	E74-like ETS transcription factor 3	2.2060	7.98E-08
1356	*CP*	ceruloplasmin	2.0245	5.12E-06
4582	*MUC1*	mucin 1, cell surface associated	1.8852	1.33E-05
Top 10 underexpressed genes				
646	*BNC1*	basonuclin 1	−3.3816	1.37E-04
316	*AOX1*	aldehyde oxidase 1	−3.1022	3.44E-05
1687	*GSDME*	gasdermin E	−2.9762	6.78E-05
65055	*REEP1*	receptor accessory protein 1	−2.6894	6.83 E-04
79804	*HAND2*	heart and neural crest derivatives expressed 2	−2.5998	1.37E-04
10332	*CLEC4M*	C-type lectin domain family 4 member M	−2.5927	2.50E-04
56245	*C21orf62*	chromosome 21 open reading frame 62	−2.5673	4.02E-05
80310	*PDGFD*	platelet derived growth factor D	−2.5513	6.75E-07
54810	*GIPC2*	GIPC PDZ domain containing family member 2	−2.5123	3.15E-09
8622	*PDE8B*	phosphodiesterase 8B	−2.5070	3.50E-04

The table listed the ten genes that were significantly up- and down-regulated according to ES after meta-analysis with *P* < 0.05.

### Gene expressional confirmation by clinical epithelial ovarian cancer samples

**Figure 3:**
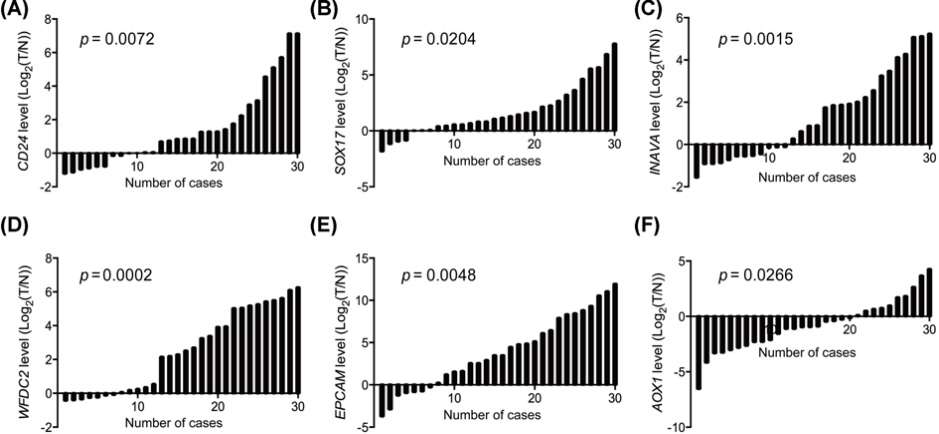
Gene expressional validation for top five up- and down-regulated genes Relative genes levels of ovarian cancer and normal tissues measured by qPCR were shown using waterfall plot. (**A–F**) Represent the expression of *CD24, SOX17, INAVA, WFDC2, EPCAM, AOX1* respectively. The fold change of relative genes expression (log2[T/N]) > 1 or < −1 was defined as significant. N, normal control; T, tumor of ovarian cancer.

In order to validate the results of DEGs for our meta-analysis, we collected 30 ovarian cancer samples and unpaired 30 normal ovarian tissues for control. RNA was extracted for each sample and the mRNA levels of each gene were quantified by real-time PCR analysis. The top five up- and down-regulated genes in ovarian cancers compared with normal control were selected for the analysis, including *CD24, SOX17, INAVA, WFDC2, EPCAM, AOX1, REEP1, BNC1, HAND2*, and *GSDME*. It has been revealed by our results that the expressional profile for the top five up-regulated genes of *CD24, SOX17, INAVA, WFDC2*, and *EPCAM* were validated to be consistent in the clinical samples with our meta-analysis (Figure 3A–E). However, only *AOX1* of top five down-regulated genes was verified to be decreased in expression for expanding sample of ovarian cancers (Figure 3F), with that of *REEP1, BNC1, HAND2*, and *GSDME* unchanged (Supplementary Figure S1A–D). The results indicated that although there are some artificial effects for the results of our meta-analysis, the assay is still of high reliability providing with genes that are potentially functional in ovarian cancer development and progress.

### WFDC2 knockdown inhibited proliferation of ovarian cancer cells

**Figure 4:**
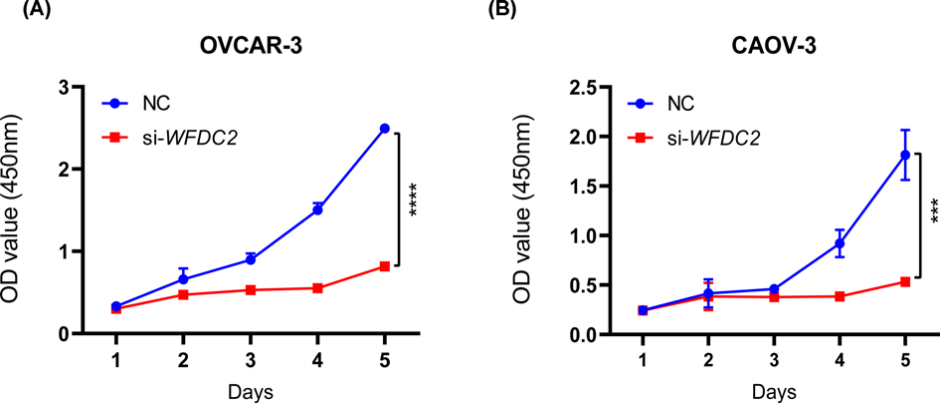
*WFDC2* knockdown inhibits proliferation of ovarian cancer cells (**A,B**) Growth curve of OVCAR-3 and CAOV-3 cells upon transfection with NC and si-*WFDC2*. Transfected cells were counted and plated in 96-well plates. ***P<0.001, ****P<0.0001.

Then, we tried to figure out whether these verified DEGs participated in the pathogenesis of ovarian cancer. Two ovarian cancer cell lines of OVCAR-3 and CAOV-3 were selected for the functional analysis. siRNAs targeting each gene were designed and synthesized by GenePharma. The knockdown efficiency for each gene was confirmed by qPCR analysis in both cells (Supplementary Figure S2A,B). So we transfected the cells with siRNAs targeting *CD24, SOX17, WFDC2, EPCAM, INAVA*, and *AOX1*, and detected cell proliferation changes by CCK8 analysis. The results showed that *WFDC2* knockdown inhibited cell proliferation in both OVCAR-3 and CAOV-3 cells (Figure 4A,B), while knockdown of *CD24, EPCAM, INAVA*, and *AOX1* leave cell proliferation largely unchanged. However, *SOX17* knockdown slightly inhibited cell proliferation of CAOV-3 cells (Supplementary Figure S3). Collectively, our data demonstrated that *WFCD2* promoted cell proliferation of ovarian cancer cells; *SOX17* might play marginal role in cell proliferation and the function is likely dependent on cellular context; *CD24, EPCAM, INAVA*, and *AOX1* had no effect on ovarian cancer proliferation.

### AOX1 knockdown decreases cell death ratio for ovarian cancer cells

**Figure 5:**
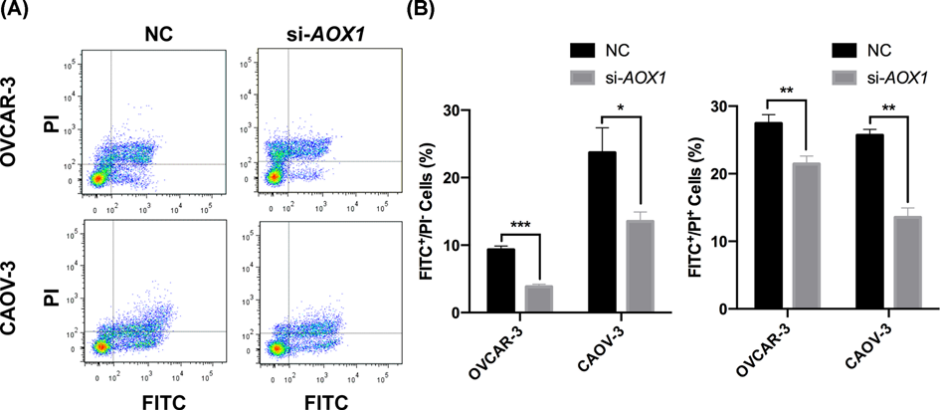
*AOX1* knockdown inhibits apoptosis of ovarian cancer cells (**A,B**) Flow cytometry analysis for apoptotic cells affected by si-*AOX1* transfection in OVCAR-3 and CAOV-3 cells. Annexin V was labeled by FITC conjugated antibodies, and the DNA was stained by Propidium Iodide. *P<0.05, **P<0.01, ***P<0.001.

Deregulated cell death is an essential characteristic of cancer cells, including ovarian cancer. Screening for genes functional in cell death regulation is always a prospective way to find targets for cancer treatment. With the hypothesis that these validated DEGs might modulate cell death of ovarian cancer cells, we detected the ratio of apoptotic cell by flow cytometry analysis. OVCAR-3 and CAOV-3 cells were transfected with siRNAs specific for these six genes, and only when *AOX1* expression was decreased, the cell death ratio will be lowered for both early (Annexin V^+^/PI^−^) and late (Annexin V^+^/PI^+^) apoptotic cells compared with NC transfected cells (Figure 5A,B). The data of cell death affected by *CD24, SOX17, WFDC2, EPCAM*, and *INAVA* knockdown were provided in Supplementary Figure S4. Collectively, these data suggested a role for *AOX1* in modulating cell death of ovarian cancer.

### INAVA and WFDC2 promote cell migration for ovarian cancer cells

**Figure 6:**
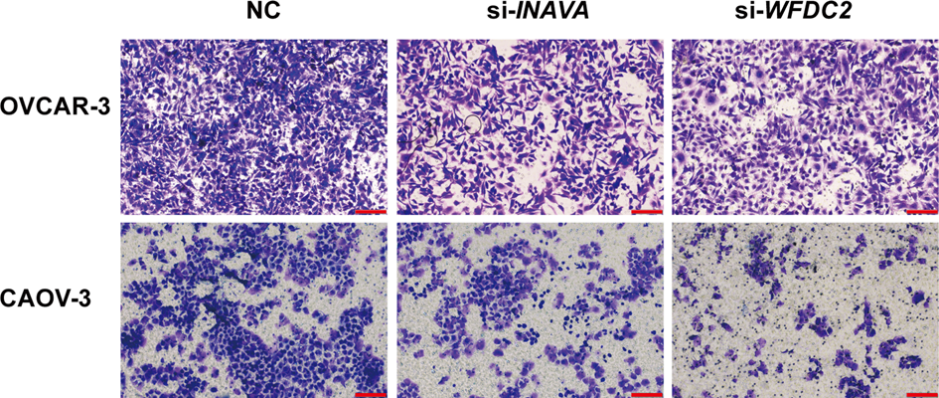
*INAVA* and *WFDC2* knockdown inhibit migration of ovarian cancer cells Down-regulation of *INAVA* and *WFDC2* in OVCAR-3 and CAOV-3 exhibited inhibition of migration (magnification, ×100). Scale bar = 100 μm. The results are representative of three separate experiments.

In addition to cell proliferation and cell death, the property of cancer cell metastasis is the most important reason for cancer recurrence and low survival rate. Cell migration assay is designed to detect whether certain genes are functional in cancer metastasis and provided with potential targets for cancer intervention. We knockdown the validated six genes in OVCAR-3 and CAOV-3 cells, and check whether cell migration ability was affected. According to our results, *WFDC2* and *INAVA* knockdown dramatically inhibited cell migration for both cell lines (Figure 6), while knockdown of *CD24, EPCAM, SOX17, AOX1* did not affected cell migration (Supplementary Figure S5). These data suggested that *WFDC2* and *INAVA* promote cell migration and might be valuable targets for cancer therapy and worth further study for the functional mechanism.

## Discussion

Despite advances in surgical and medical therapy, the overall survival rate for patients with ovarian cancer remains largely unchanged over the past decades. The lethality of ovarian cancer is mainly due to the difficulties in diagnosis at early stage and the lack of effective treatments for patients with an advanced status [27]. Since different ovarian cancer cells are biologically, genetically, and molecularly similar, identification of common molecular markers may contribute to reveal the appropriate risk factors, as well as make preventive, diagnostic, and therapeutic decisions for these patients. Recently, microarray technology has been developing rapidly and widely used to uncover the general genetic alterations in progression of diseases, which enables the identification of targets for diagnosis, therapeutic, and prognosis of tumors [28]. Despite significant amounts of studies using microarray-based technology to identify molecular markers in different ovarian cancer cells, contradictory results have been reported due to the diversity in patient selection, tissue source, and study designs [29]. Therefore, in order to recognize the common gene signatures underlying ovarian cancer, we performed meta-analysis by using 16 publicly available microarray datasets. In the present study, 972 DEGs were found at with *P*-value < 0.001, including 541 up- and 431 down-regulated, respectively. Interestingly, by comparing the list of meta-based DEGs and that of individual microarray dataset, 92 additional DEGs were found as gained DEGs, which means our meta-based analysis lead to discovery of new DEGs compared with individual dataset. By uncovering shared gene expressional profiles, the present study highlighted potential diagnostic and therapeutic biomarkers in ovarian cancer, and might aid in understanding the molecular mechanisms of its development and progression.

Among the up-regulated DEGs, the expression for top five genes were further validated in clinical samples, including *CD24, EPCAM, SOX17, WFDC2*, and *INAVA*. The protein encoded by *CD24* is a small, heavily glycosylated mucin-like cell surface protein [30]. Accumulating evidences had demonstrated that CD24 was involved in epithelial–mesenchymal transition (EMT) and its increased expression was correlated with poor prognosis in patients of epithelial ovarian cancer [31,32]. *EPCAM*, an epithelia cell localized type I transmembrane glycoprotein, was initially identified as a highly expressed tumor-associated antigen in ovarian cancer [33,34]. Collectively, these reported data proved the accuracy for our meta-analysis based DEGs. *SOX17*, an important member of SOX family, which encodes an antagonist of Wnt signaling pathway, was found to be frequently methylated in high-grade serous ovarian cancer [35,36]. It is well known that promoter methylation typically represses gene expression. Contradictory, *SOX17* expression was revealed to be up-regulated in our meta-analysis and clinical samples. So the expressional profiles and relevance of *SOX17* in ovarian cancer remains to be further validated. *WFDC2* is located on human chromosome 20q12-13.1, the protein product for which is also called human epididymis protein 4 (HE4). It has been reported that WFDC2 is involved in epithelial–mesenchymal transformation, and this gene is highly expressed in epithelial ovarian cancer than in normal epithelial cells [37,38]. Besides, our functional study on WFDC2 effects on cell proliferation consolidated the previous discovery that *WFDC2* gene overexpression could promote ovarian tumor growth [39]. Thus, targeted screening for WFDC2 protein inhibition might benefits for the treatment of ovarian cancer. *INAVA* gene, also called *C1orf106*, has been recently reported essential for optimal MAPK and NF-κB activation in primary human cells by recruiting 14-3-3τ [40,41]. And inflammatory bowel disease (IBD)-associated polymorphisms in *INAVA* is revealed to play important roles in intestinal immune homeostasis by regulating pattern recognition receptor (PRR)-mediated signaling transduction and bacterial clearance. In our analysis, *INAVA* was found to promote migration of ovarian cancer cells, which unmask a novel role for *INAVA* in ovarian cancer progression and pointed potential implication for *INAVA* in targeted ovarian cancer treatment.

For the down-regulated DEGs, expressional confirmation was also performed in the clinical samples for the top five dysregulated genes of *AOX1, REEP1, BNC1, HAND2*, and *GSDME*. However, only *AOX1* was verified with consistent expressional profile with the result of our meta-analysis, which implied that artificial effects for meta-analysis existed and could be eliminated by further validation. *AOX1* is a xenobiotic enzyme, which is a family member of xanthine oxidase of cytosolic molybdoenzymes [42]. The enzyme catalyzes the oxidation of aldehydes and aromatic N-heterocycles [43]. Recently, *AOX1* has been uncovered to participate in the development and progression of a series of cancers. For example, loss of *AOX1* expression by epigenetic regulation could promote bladder cancer via metabolic deregulations; the SNP rs73055188 of *AOX1* locus is related to prostate cancer survival time, and *AOX1* gene expression level is correlated with recurrence of prostate cancer [44]. However, the roles for *AOX1* in ovarian cancer have not been addressed [45]. In this study, we found that knockdown of *AOX1* in ovarian cancer cells inhibited cell apoptosis. Although the mechanism underlying which *AOX1* functions in ovarian cancer has not been elucidated, the therapeutic value for AOX1 is still worth further exploration.

## Conclusions

In our study, several DEGs, including *WFDC2, INAVA*, and *AOX1*, in ovarian cancer revealed by our meta-analysis have been validated to potentially participate in ovarian cancer development and progression. Importantly, our study will shed light on the novel molecular mechanisms underlying the pathogenesis of ovarian cancer, and facilitate the understanding of novel diagnostic and therapeutic targets in ovarian cancer.

## Supplementary Material

Supplementary Figures S1-S5 and Tables S1-S3

## Data Availability

The data that support the findings of the present study are available within the article or on reasonable request from the corresponding author.

## Abbreviations

*AOX1*: aldehyde oxidase 1
BMP: bone morphogenetic protein
*BNC1*: basonuclin 1
CCK8: cell counting kit-8
*CD24*: CD24 molecule
DEG: differentially expressed gene
*EPCAM*: epithelial cell adhesion molecule
ES: effect size
FBS: fetal bovine serum
GAPDH: glyceraldehyde-3-phosphate dehydrogenase
GEO: Gene Expression Omnibus
*GSDME*: gasdermin E
*HAND2*: heart and neural crest derivatives expressed 2
*INAVA*: innate immunity activator
PCA: principal component analysis
*REEP1*: receptor accessory protein 1
REM: random effect modeling
SNP: single nucleotide polymorphism
*SOX17*: SRY (sex determining region Y)-box transcription factor 17
TGFβ: transforming growth factor-β
WFDC2: WAP four disulfide core domain 2
