# Supplementary material for: Meta-analysis based gene expression profiling reveals functional genes in ovarian cancer
Source: Biosci Rep. 2020 Nov 19;40(11):BSR20202911. doi: 10.1042/BSR20202911 (PMC7677829; doi:10.1042/BSR20202911)
Supplement: Supplementary Figures S1-S5 and Tables S1-S3 [file BSR-2020-2911_supp.pdf]

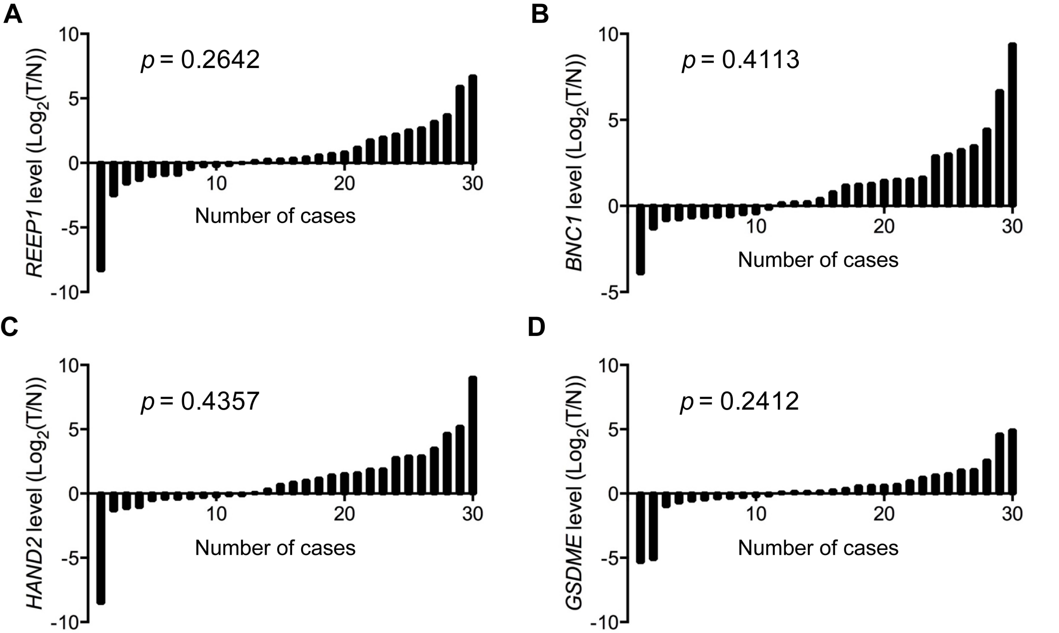
A
B
C
D
p = 0.2642
p = 0.4113
p = 0.4357
p = 0.2412
REEP1 level (Log2(T/N))
BNC1 level (Log2(T/N))
HAND2 level (Log2(T/N))
GSDME level (Log2(T/N))
Number of cases
Number of cases
Number of cases
Number of cases

Supplementary Figure 1. Four Down-regulated genes fail to be validated by extended samples. Relative gene levels of ovarian cancer and normal tissues measured by qPCR were shown using waterfall plot. **(A-D)** Represent the levels of *REEP1, BNC1, HAND2, GSDME* separately.

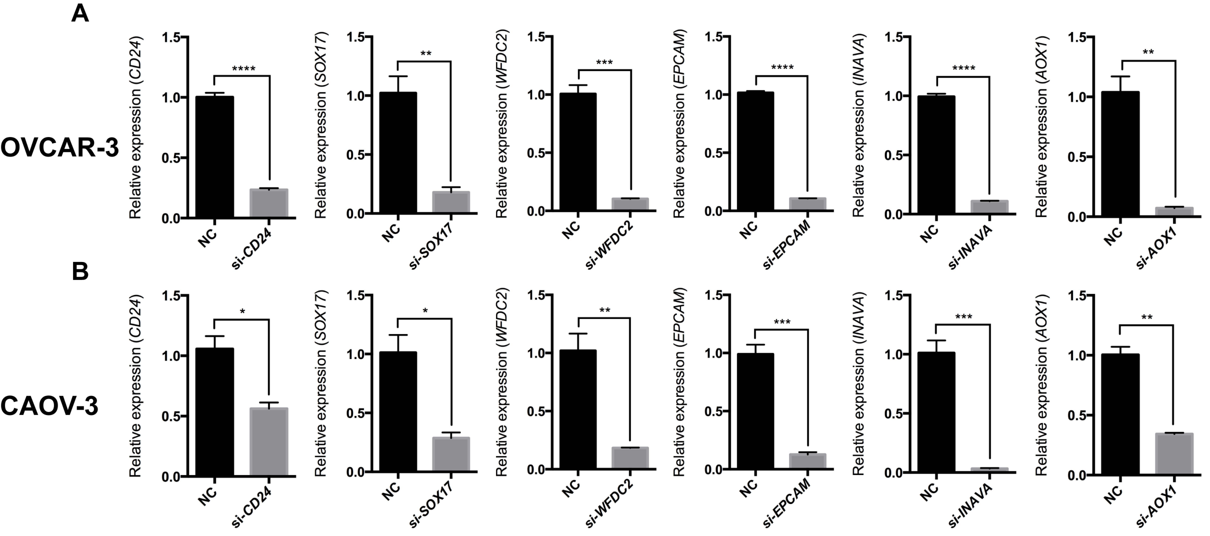
A
B
OVCAR-3
CAOV-3
Relative expression (CD24)
Relative expression (SOX17)
Relative expression (WFDC2)
Relative expression (EPCAM)
Relative expression (INAVA)
Relative expression (AOX1)
1.5
1.0
0.5
0.0
NC
si-CD24
si-SOX17
si-WFDC2
si-EPCAM
si-INAVA
si-AOX1
****
**
***
*

Supplementary Figure 2. Gene knockdown efficiency. (A-B) QPCR analysis for the expression of each gene in OVCAR-3 and CAOV-3 cells upon NC, si-*CD24*, si-*SOX17*, si-*WFDC2*, si-*EPCAM*, si-*INAVA* and si-*AOX1* transfection.

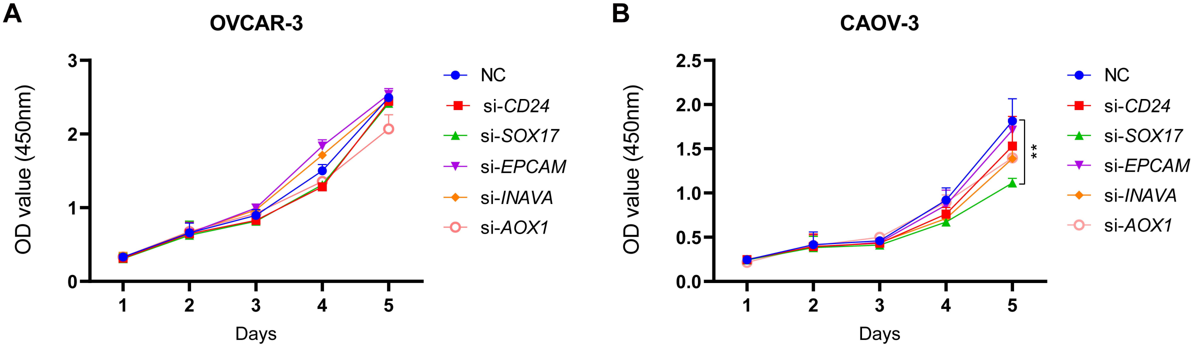
A
OVCAR-3
OD value (450nm)
3
2
1
0
1
2
3
4
5
Days
NC
si-CD24
si-SOX17
si-EPCAM
si-INAVA
si-AOX1
B
CAOV-3
OD value (450nm)
2.5
2.0
1.5
1.0
0.5
0.0
1
2
3
4
5
Days
**
NC
si-CD24
si-SOX17
si-EPCAM
si-INAVA
si-AOX1

Supplementary Figure 3. Genes that have no effects on proliferation for ovarian cancer cells. (A-B) Growth curve of OVCAR-3 and CAOV-3 cells upon transfection with NC and si-*CD24,* si-*SOX17,* si-*EPCAM,* si-*INAVA* and si-*AOX1*. Transfected cells were counted and plated in 96-well plates.

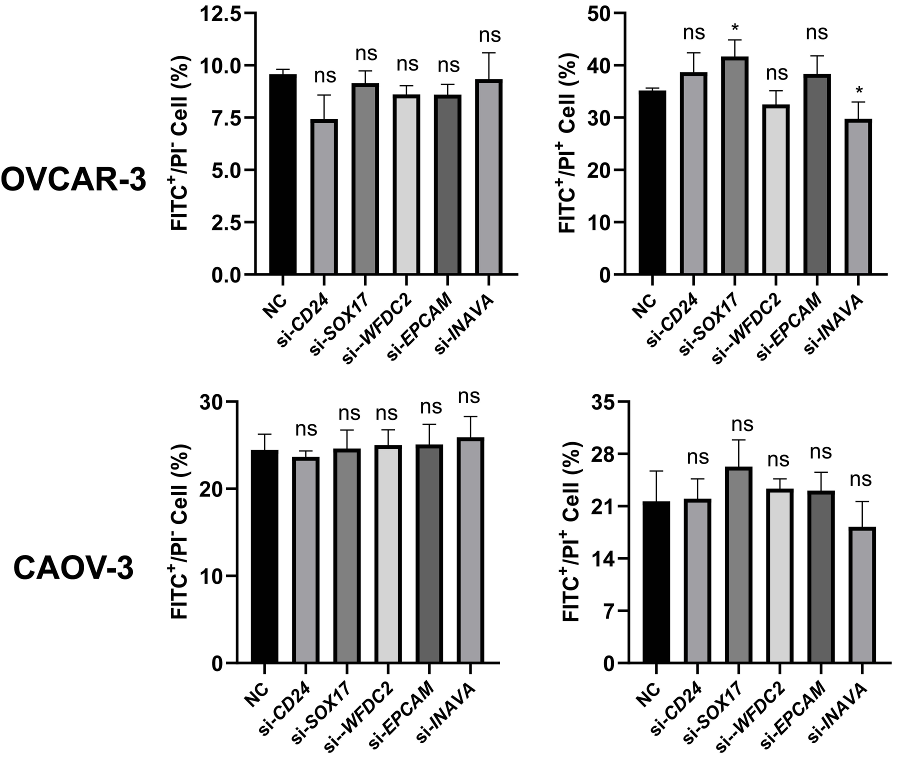
OVCAR-3
CAOV-3
FITC+/PI- Cell (%)
FITC+/PI+ Cell (%)
12.5
10.0
7.5
5.0
2.5
0.0
50
40
30
20
10
0
30
20
10
35
28
21
14
7
ns
*
NC
si-CD24
si-SOX17
si--WFDC2
si-EPCAM
si-INAVA

Supplementary Figure 4. Genes that have no effects on apoptosis for ovarian cancer cells. NC, si-*CD24,* si-*SOX17,* si-*WFDC2,* si-*EPCAM* and si-*INAVA* were transfected into OVCAR-3 and CAOV-3 cells, cell apoptosis analysis was performed as indicated in methods.

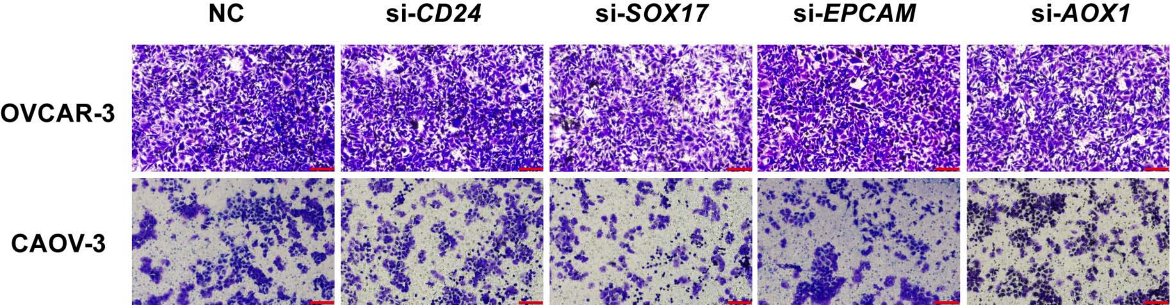
NC
si-*CD24*
si-*SOX17*
si-*EPCAM*
si-*AOX1*
OVCAR-3
CAOV-3

Supplementary Figure 5. Genes that have no effects on migration for ovarian cancer cells. Transwell assay analyzed the migration of OVCAR-3 and CAOV-3 upon NC, si-*CD24,* si-*SOX17,* si-*EPCAM,* si-*INAVA* and si-*AOX1* transfection (magnification, ×100). Scale bar= 100 μ m.

Supplementary Table1. The clinicopathologic features of ovarian cancer patients.

Characteristics		Total number
Age	< 50	12
	≥ 50	18
Tumor grades	I-II	11
	III	19
Tumor stage	I-II	13
	III-IV	17

The table lists the age and tumor grade and tumor stage of the 30 ovarian cancer patients used in the study.

Supplementary Table 2. Oligo sequences used in the experiments.

Oligo ID	Sequence (5’-3’)
Negative control	Sense: UUCUCCGAACGUGUCACGUTT<br>Antisense: ACGUGACACGUUCGGAGAATT
CD24-siRNA	Sense: GCACUAAUUUAAUGCCGAUTT<br>Antisense: AUCGGCAUUAAAUUAGUGCTT
SOX17-siRNA	Sense: CACGGAAUUUGAACAGUAUTT<br>Antisense: AUACUGUUCAAAUUCCGUGTT
EPCAM-siRNA	Sense: GGAUCAUCAUUGAACUAAATT<br>Antisense: UUUAGUUCAAUGAUGAUCCTT
WFDC2-siRNA	Sense: ACCAGAACUGCACGCAAGATT<br>Antisense: UCUUGCGUGCAGUUCUGGUTT
INAVA-siRNA	Sense: GCAAGUCUUUGUACCUGAATT<br>Antisense: UUCAGGUACAAAGACUUGCTT
AOX1-siRNA	Sense: GCAGCCAAAUGUGGACUAUTT<br>Antisense: AUAGUCCACAUUUGGCUGCTT

The sequences of siRNAs used for CD24, SOX17, WFDC2, EPCAM, INAVA, BNC1, AOX1, GSDME, REEP1, and HAND2 knockdown are listed in this table .

Supplementary Table 3. List of PCR primer sequences used in mRNA expression analysis.

Gene	Species	Sequence (5’-3’)
GAPDH	Homo sapiens	Forward: GGAGCGAGATCCCTCCAAAAT<br>Reverse: GGCTGTTGTCATACTTCTCATGG
CD24	Homo sapiens	Forward: AGAGATAACCCTGCCCGAGG<br>Reverse: CCCCCAAAAGAAAAGTCCGC
SOX17	Homo sapiens	Forward: CAAGGGCGAGTCCCGTATCC<br>Reverse: CGACTTGCCCAGCATCTTGC
EPCAM	Homo sapiens	Forward: AGCGAGTGAGAACCTACTGGA<br>Reverse: CGCGTTGTGATCTCCTTCTGA
WFDC2	Homo sapiens	Forward: TGTTCGGCTTCACCCTAGTC<br>Reverse: TCCTTATCATTGGGCAGAGAGC
INAVA	Homo sapiens	Forward: GCGGAGTATCCCCTCAAACC<br>Reverse:TTAGGGGGTCCTCTCTGTGC
BNC1	Homo sapiens	Forward: CAGCGGTCGCAGGATGG<br>Reverse:AGCTTACTTAGAGCGTGGGC
AOX1	Homo sapiens	Forward: TCATCTAAGGGTCTGGGAGAGT<br>Reverse:TCCAGGTTCATCTCTCGGAA
GSDME	Homo sapiens	Forward: CAGCTTACAGGGTGGGTGTC<br>Reverse:GCGCTATCTGGCATTTCTGC
REEP1	Homo sapiens	Forward: GATGGCTGCTTCCAAGGGAC<br>Reverse:TCTTAGGCTGGCCGTGTTTG
HAND2	Homo sapiens	Forward: GCCTCCTGGAAGAAGATCCA<br>Reverse: GCCAAGACACCCTGTAGAGC

The sequences of primers used for CD24, SOX17, WFDC2, EPCAM, INAVA, BNC1, AOX1, GSDME, REEP1, and HAND2 are listed in this table.
